# Prognostic value of fibrinogen in patients with coronary artery disease and prediabetes or diabetes following percutaneous coronary intervention: 5-year findings from a large cohort study

**DOI:** 10.1186/s12933-021-01335-1

**Published:** 2021-07-16

**Authors:** Deshan Yuan, Ping Jiang, Pei Zhu, Sida Jia, Ce Zhang, Yue Liu, Ru Liu, Jingjing Xu, Xiaofang Tang, Xueyan Zhao, Runlin Gao, Yuejin Yang, Bo Xu, Zhan Gao, Jinqing Yuan

**Affiliations:** State Key Laboratory of Cardiovascular Disease, Fuwai Hospital, National Center for Cardiovascular Diseases, Chinese Academy of Medical Sciences and Peking Union Medical College, No 167, Beilishi Road, Xicheng District, Beijing, 100037 China

**Keywords:** Fibrinogen, Coronary artery disease, Percutaneous coronary intervention, Prediabetes, Diabetes

## Abstract

**Background:**

Fibrinogen (FIB) is an independent risk factor for mortality and cardiovascular events in the general population. However, the relationship between FIB and long-term mortality among CAD patients undergoing PCI remains unclear, especially in individuals complicated with diabetes mellitus (DM) or prediabetes (Pre-DM).

**Methods:**

6,140 patients with CAD undergoing PCI were included in the study and subsequently divided into three groups according to FIB levels (FIB-L, FIB-M, FIB-H). These patients were further grouped by glycemic status [normoglycemia (NG), Pre-DM, DM]. The primary endpoint was all-cause mortality. The secondary endpoint was cardiac mortality.

**Results:**

FIB was positively associated with hemoglobin A1c (HbA1c) and fasting blood glucose (FBG) in CAD patients with and without DM (P < 0.001). During a median follow-up of 5.1 years (interquartile range 5.0–5.2 years), elevated FIB was significantly associated with long-term all-cause mortality (adjusted HR: 1.86; 95% CI 1.28–2.69; P = 0.001) and cardiac mortality (adjusted HR: 1.82; 95% CI 1.15–2.89; P = 0.011). Similarly, patients with DM, but not Pre-DM, had increased risk of all-cause and cardiac mortality compared with NG group (all P < 0.05). When grouped by both FIB levels and glycemic status, diabetic patients with medium and high FIB levels had higher risk of mortality [(adjusted HR: 2.57; 95% CI 1.12–5.89), (adjusted HR: 3.04; 95% CI 1.35–6.82), all P < 0.05]. Notably, prediabetic patients with high FIB also had higher mortality risk (adjusted HR: 2.27; 95% CI 1.01–5.12).

**Conclusions:**

FIB was independently associated with long-term all-cause and cardiac mortality among CAD patients undergoing PCI, especially in those with DM and Pre-DM. FIB test may help to identify high-risk individuals in this specific population.

**Supplementary Information:**

The online version contains supplementary material available at 10.1186/s12933-021-01335-1.

## Background

Despite advances in revascularization strategies over recent decades, the clinical outcomes remain unfavorable in patients with coronary artery disease (CAD), especially when complicated with diabetes mellitus (DM)^1^. Regarded as a pivotal component of coagulation as well as a biomarker of inflammation, fibrinogen (FIB) plays a crucial role in the pathophysiological process of thrombosis and atherosclerosis^2^–^5^. Previous evidences suggested that FIB was an independent risk factor of CAD development and cardiovascular events in the general population^6^,^ 7^. Similar results on the prognostic value of FIB were also observed in patients with CAD^8^–^10^.

Glycemic metabolism abnormality, including DM and prediabetes (pre-DM), is increasingly prevalent on a global scale. By 2045, over 600 million individuals are projected to develop DM, with about the same number developing pre-DM^11^. The cardiovascular disease (CVD) risk, disability and mortality brought by glycemic metabolism abnormality is undisputedly a serious public health concern. Interestingly, FIB level was found to be higher in diabetic and prediabetic patients, and was involved in glycemic metabolism abnormality and insulin resistance^12^,^ 13^. Moreover, recent studies reported that FIB was positively related with the glycemic metabolism (hemoglobin A1c [HbA1c] and fasting blood glucose [FBG]) in patients with acute coronary syndrome (ACS) or stable CAD^9^,^ 10^. However, few data are available examining the correlation between FIB and glycemic metabolism in CAD patients undergoing percutaneous coronary intervention (PCI). Furthermore, the association of FIB with long-term outcomes in this population was far less investigated, particularly in those with impaired glycemic metabolism.

In light of the above, we aimed to evaluate the relationship between FIB and glycemic metabolism, and further determine the combined effect of FIB and impaired glycemic metabolism on long-term all-cause and cardiac mortality in CAD patients undergoing PCI.

## Methods

### Study population

**Fig. 1:**
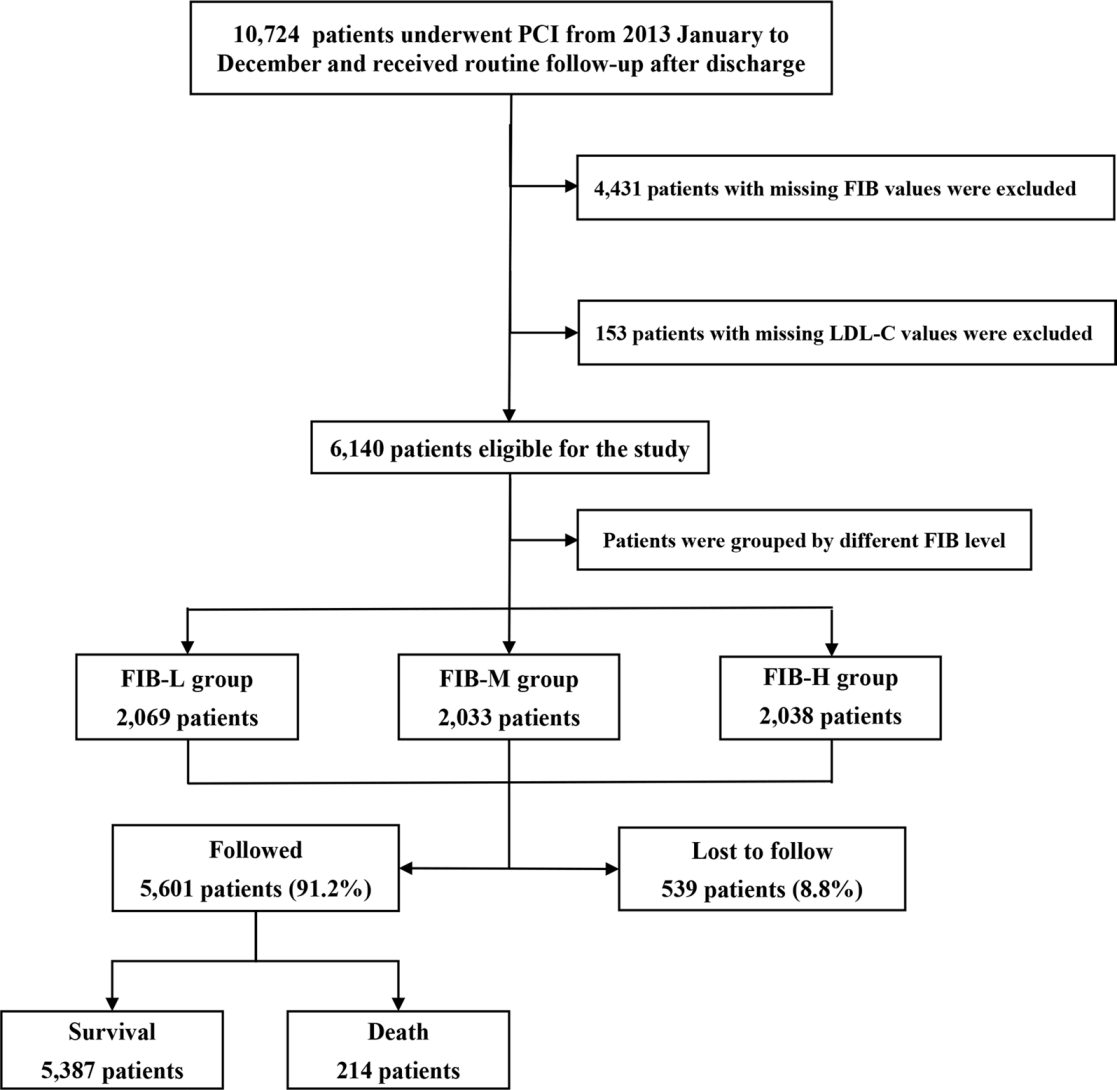
Study flowchart. FIB, fibrinogen, LDL-C, low-density lipoprotein cholesterol, PCI, percutaneous coronary intervention

This study was based on a prospective, observational, single-center cohort. From January 2013 to December 2013, 10,724 CAD patients were consecutively enrolled undergoing PCI at Fuwai Hospital, Chinese Academy of Medical Sciences (Beijing, China) (Fig. 1). Patients with missing FIB (n = 4431) and Low-density lipoprotein cholesterol (n = 153) values were excluded. A total of 6,140 patients were ultimately included in the analysis. The study protocol was approved by the Institutional Review Board of Fuwai Hospital and complied with the Declaration of Helsinki. All patients provided written informed consent before the intervention. Regular follow-up assessment of patients was performed at five time points (1-month, 6-month, 12-month, 2-year, and 5-year after the discharge). Follow-up data were collected through medical records and telephone interview. The primary endpoint was all-cause mortality. The secondary outcome was cardiac mortality. Mortality that could not be attributed to a noncardiac etiology was considered cardiac mortality. All endpoints were adjudicated centrally by 2 independent cardiologists, and disagreement was resolved by consensus.

### Procedure and medications

Before the procedure, patients receiving selective PCI were treated with aspirin (300 mg) and ticagrelor (loading dose, 180 mg) or clopidogrel (loading dose, 300 mg), except for patients already on dual antiplatelet therapy; for patients with ACS receiving emergency PCI, the same dose of aspirin and ticagrelor or clopidogrel (loading dose, 300–600 mg) were administered as soon as possible. All patients were administered with unfractionated heparin (100 U/kg), and interventional cardiologist decided whether to use glycoprotein IIb/IIIa antagonist according to the clinical conditions and coronary lesions during the procedure. After the procedure, the dual antiplatelet therapy including aspirin (100 mg daily), ticagrelor (90 mg, twice daily) or clopidogrel (75 mg, daily) were recommended for at least 1 year. The choice of equipment and techniques during PCI was at the discretion of the physicians.

### Definition of clinical status

Glycemic categories were based on the guideline recommendations of American Diabetes Association^14^. Diabetes mellitus (DM) was defined by HbA1c levels ≥ 6.5%, or fasting blood glucose (FBG) ≥ 7.0 mmol/L, or 2-h blood glucose levels of oral glucose tolerance test ≥ 11.1 mmol/L), or current use of hypoglycemic medications. Prediabetes (Pre-DM) was defined as nondiabetic patients with FBG ranging from 5.6 to 6.9 mmol/L, HbA1c levels ranging from 5.7% to 6.4%. Patients without Pre-DM or DM were defined as normoglycemia (NG). Hypertension was defined as systolic blood pressure ≥ 140 mmHg and/or diastolic blood pressure ≥ 90 mmHg and/or current use of antihypertensive drugs. Low-density lipoprotein cholesterol ≥ 3.4 mmol/L, fasting total cholesterol ≥ 5.2 mmol/L, triglyceride ≥ 1.7 mmol/L, high-density lipoprotein cholesterol < 1.0 mmol/L and/or chronic use of lipid-lowering drugs were considered criteria for dyslipidemia. Left main disease was defined as stenosis of ≥ 50% in left main coronary artery, and three-vessel disease was defined as stenosis of ≥ 50% in all three main coronary arteries (right coronary artery, left circumflex artery and left anterior descending artery) confirmed by coronary angiography.

### Laboratory analysis

Fasting blood samples were drawn from all patients within 24 h after admission. Enzymatic hexokinase method was used to measure the concentrations of blood glucose. Tosoh Automated Glycohemoglobin Analyzer (HLC-723G8) was used to measure the HbA1c levels. Stago autoanalyzer with the STA fibrinogen kit (Diagnostica Stago, Taverny, France) was used to measure the concentrations of FIB. All other laboratory measurements were conducted at the biochemistry center of Fu Wai Hospital by standard biochemical techniques.

### Statistical analysis

Continuous variables were presented as mean ± standard deviation, while categorical variables were presented as frequency and percentage. Differences of continuous and categorical variables were analyzed by analysis of variance or Kruskal–Wallis test and χ^2^ test or Fisher's exact test, as appropriate. Pearson correlation and linear regression analysis were performed to evaluate the correlation between FIB and glycemic metabolism (HbA1c and FBG). In survival analysis, the association between FIB and clinical endpoint was initially examined using restricted cubic splines. The FIB was subsequently analyzed as both a continuous and a categorical variable. For categorical analysis, patients were grouped according to tertiles of the distribution [FIB-L(< 2.98 mg/dL), FIB-M(2.98–3.59 mg/dL), FIB-H(< 3.59 mg/dL)]. Survival distributions were presented by Kaplan–Meier curves and compared by log-rank test. Cox regression analyses were performed to calculate the hazard ratios (HRs) and 95% confidence interval (CI). Proportional hazards assumption was verified by Schoenfeld residuals. In multivariate Cox analyses, covariates including age, sex, body mass index (BMI), hypertension, family history of CAD, prior PCI/CABG, LVEF, LDL-C, creatine, DES implantation, clopidogrel, ACEI/ARB were adjusted because of their statistical significance in univariate analysis or clinical importance. The prognostic impact of glycemic metabolism status (NG, Pre-DM and DM) for all-cause mortality was also assessed by using the model mentioned above. Patients were further divided into 9 groups by both FIB levels and glycemic metabolism status to calculate HRs for all-cause mortality using FIB-L plus NG as reference. Statistical analyses were conducted with SPSS version 25.0 (IBM Corp., Armonk, N.Y., USA), R Programming Language version 4.0.0 (R Core Team, 2014), and GraphPad Prism version 7.0.0 for windows (GraphPad Software, San Diego, California USA). A two-tailed P value < 0.05 was considered statistically significant.

## Results

### Baseline characteristics of patients with different FIB levels

Among the 6,140 patients, the mean age was 58.4 ± 10.4 years, and 4771(77.7%) were male. The baseline characteristics of patients according to the tertiles of FIB are summarized in Table 1. Patients with higher FIB levels were older and less likely to be male (all P < 0.05). In addition, they had higher proportion of diabetes, hypertension, prior stroke, ACS, and left main or three-vessel disease (all P < 0.05). Moreover, patients with elevated FIB levels had higher FBG, HbA1c, hs-CRP, D-dimer, TC, LDL cholesterol, creatinine, lesion vessels, SYNTAX score, but lower LVEF and lower rate of complete revascularization (all P < 0.05). No significant differences were noted regarding dyslipidemia, family history of CAD, smoking, HDL cholesterol, number of stents and DES implantation among these groups (all P > 0.05).

**Table 1:** Baseline characteristics for patients with different FIB levels

Variables	Total (n = 6140)	FIB-L (n = 2069)	FIB-M (n = 2033)	FIB-H (n = 2038)	*P* value
Age, years	58.4 ± 10.4	57.3 ± 10.2	58.2 ± 10.2	59.6 ± 10.6	< 0.001
Male, %	4771 (77.7)	1729(83.6)	1567(77.1)	1475(72.4)	< 0.001
BMI, kg/m^2^	25.9 ± 3.2	25.7 ± 3.1	26.1 ± 3.2	25.9 ± 3.3	< 0.001
Diabetes mellitus, %	1862 (30.3)	521(25.2)	656(32.3)	685(33.6)	< 0.001
Hypertension, %	3930 (64.0)	1248(60.3)	1328(65.3)	1354(66.4)	< 0.001
Dyslipidemia, %	4241 (69.1)	1403(67.8)	1436(70.6)	1402(68.8)	0.140
Family history of CAD, %	1433 (23.3)	501(24.2)	479(23.6)	453(22.2)	0.309
Smoking, %	3496 (56.9)	1197(57.9)	1173(57.7)	1126(55.3)	0.169
Prior MI, %	1219 (19.9)	465(22.5)	394(19.4)	360(17.7)	< 0.001
Prior PCI or CABG, %	1581 (25.7)	621(30.0)	514(25.3)	446(21.9)	< 0.001
Prior stroke, %	658 (10.7)	204(9.9)	206(10.1)	248(12.2)	0.033
Clinical presentation, %					< 0.001
SAP	2572 (41.9)	995 (48.1)	874 (43.0)	703 (34.5)	
ACS	3568 (58.1)	1074 (51.9)	1159 (57.0)	1335 (65.5)	
LVEF, %	63.0 ± 7.2	63.6 ± 6.8	63.5 ± 6.8	62.0 ± 7.9	< 0.001
FBG, mmol/L	6.1 ± 2.0	5.8 ± 1.8	6.1 ± 2.0	6.3 ± 2.2	< 0.001
HbA1c, %	6.6 ± 1.2	6.4 ± 1.0	6.6 ± 1.2	6.8 ± 1.4	< 0.001
FIB, mg/dL	3.40 ± 0.84	2.62 ± 0.28	3.26 ± 0.17	4.32 ± 0.74	< 0.001
hs-CRP, mg/L	3.14 ± 3.77	1.31 ± 1.74	2.34 ± 2.65	5.81 ± 4.64	< 0.001
D-dimer, μg/mL	0.41 ± 0.65	0.37 ± 0.70	0.38 ± 0.52	0.48 ± 0.70	< 0.001
TC, mmol/L	4.17 ± 1.09	4.00 ± 1.07	4.21 ± 1.08	4.30 ± 1.11	< 0.001
HDL-C, mmol/L	1.02 ± 0.28	1.02 ± 0.27	1.03 ± 0.28	1.00 ± 0.28	0.060
LDL-C, mmol/L	2.48 ± 0.92	2.34 ± 0.91	2.50 ± 0.91	2.59 ± 0.93	< 0.001
TG, mmol/L	1.81 ± 1.12	1.75 ± 1.12	1.85 ± 1.13	1.83 ± 1.12	0.015
Creatinine (μmol/L)	75.4 ± 16.0	74.5 ± 14.1	74.6 ± 15.4	77.0 ± 18.2	< 0.001
Lesion vessels	1.4 ± 0.7	1.4 ± 0.6	1.4 ± 0.7	1.5 ± 0.7	< 0.001
LM/three-vessel disease, %	2773 (45.2)	865 (41.8)	909 (44.7)	999 (49.0)	< 0.001
SYNTAX score^a^	12.0 ± 8.2	11.2 ± 7.8	11.9 ± 8.2	12.8 ± 8.5	< 0.001
Complete revascularization, %	3310 (53.9)	1201 (58.0)	1090 (53.6)	1019 (50.0)	< 0.001
Number of stents	1.8 ± 1.2	1.8 ± 1.1	1.8 ± 1.2	1.9 ± 1.2	0.148
DES implantation, %	5778 (94.1)	1941 (93.8)	1923 (94.6)	1914 (93.9)	0.520
Medications at discharge, %					
Aspirin	6077 (99.0)	2051(99.1)	2011(98.9)	2015(98.9)	0.680
Clopidogrel	6020 (98.0)	2023(97.8)	1997(98.2)	2000(98.1)	0.542
β-blocker	5609 (91.4)	1878(90.8)	1868(91.9)	1863(91.4)	0.443
ACEI/ARB	3150 (51.3)	983(47.5)	1049(51.6)	1118(54.9)	< 0.001
CCB	2875 (46.8)	952(46.0)	973(47.9)	950(46.6)	0.482
Statin	5923 (96.5)	2002(96.8)	1963(96.6)	1958(96.1)	0.473
Nitrate	5984 (97.5)	2021(97.7)	1981(97.4)	1982(97.3)	0.683

Values are presented as mean ± standard deviation or number (%)

*ACEI* angiotensin-converting enzyme inhibitors, *ACS* acute coronary syndrome, *ARB* angiotensin II receptor blockers, *BMI* body mass index, *CAD* coronary artery disease, *CABG* coronary artery bypass grafting, *CCB* calcium channel blocker, *DES* drug-eluting stent, *FIB* fibrinogen, *FBG* fasting blood glucose, *hs-CRP* high sensitivity C-reactive protein, *HbA1c* Hemoglobin A1c, *HDL-C* high-density lipoprotein cholesterol, *LDL-C* low-density lipoprotein cholesterol, *LM* left main disease, *LVEF* left ventricular ejection fraction, *MI* myocardial infarction, *PCI* percutaneous coronary intervention, *SAP* stable angina pectoris, *TC* total cholesterol, *TG* triglycerides

^a^Calculated using an online calculator (http://www.syntaxscore.com/) by a dedicated research group blinded to the clinical data

### Comparison of clinical data among groups with different glycemic metabolism status

In Table 2, Patients were divided into three subgroups based on different glycemic metabolism status. In general, the DM and pre-DM group had a less favorable cardiovascular risk profile. Patients with DM or pre-DM tended to be older and female, with a larger burden of concomitant diseases, such as hypertension, dyslipidemia and prior stroke compared with those in NG group (all P < 0.05). Additionally, the prevalence of prior PCI/CABG and left main or three-vessel disease was higher in the DM and pre-DM group (all P < 0.05). Meanwhile, there were also higher BMI, FBG, HbA1c, TG, number of diseased vessels, SYNTAX score, and lower LVEF, HDL cholesterol, complete revascularization, DES implantation in the DM group (all P < 0.05). Notably, FIB levels were significantly elevated from NG to DM group (P < 0.001).

**Table 2:** Baseline characteristics for patients with different glycemic metabolism status

Variables	Total (n = 6140)	NG (n = 1394)	Pre-DM (n = 2884)	DM (n = 1862)	*P* value
Age, years	58.4 ± 10.4	55.9 ± 10.8	58.9 ± 10.4	59.4 ± 9.8	< 0.001
Male, %	4771 (77.7)	1138(81.6)	2224(77.1)	1409(75.7)	< 0.001
BMI, kg/m^2^	25.9 ± 3.2	25.7 ± 3.1	25.8 ± 3.2	26.2 ± 3.2	< 0.001
Hypertension, %	3930 (64.0)	830(59.5)	1791(62.1)	1309(70.3)	< 0.001
Dyslipidemia, %	4241 (69.1)	906(65.0)	1910(66.2)	1425(76.5)	< 0.001
Family history of CAD, %	1433 (23.3)	355(25.5)	653(22.6)	425(22.8)	0.101
Smoking, %	3496 (56.9)	810(58.1)	1664(57.7)	1022(54.9)	0.098
Prior MI, %	1219 (19.9)	265(19.0)	567(19.7)	387(20.8)	0.426
Prior PCI or CABG, %	1581 (25.7)	322(23.1)	729(25.3)	530(28.5)	0.002
Prior stroke, %	658 (10.7)	130(9.3)	279(9.7)	249(13.4)	< 0.001
Clinical presentation, %					< 0.001
SAP	2572 (41.9)	537 (38.5)	1193 (41.4)	842 (45.2)	
ACS	3568 (58.1)	857 (61.5)	1691 (58.6)	1020 (54.8)	
LVEF, %	63.0 ± 7.2	63.0 ± 7.2	63.3 ± 7.1	62.6 ± 7.4	< 0.001
FBG, mmol/L	6.1 ± 2.0	4.7 ± 0.4	5.2 ± 0.6	7.3 ± 2.5	0.009
HbA1c, %	6.6 ± 1.2	5.4 ± 0.2	6.0 ± 0.2	7.5 ± 1.3	< 0.001
FIB, mg/dL	3.40 ± 0.84	3.32 ± 0.85	3.39 ± 0.85	3.47 ± 0.82	< 0.001
hs-CRP, mg/L	3.14 ± 3.77	3.06 ± 3.82	3.11 ± 3.71	3.26 ± 3.82	0.260
D-dimer, μg/mL	0.41 ± 0.65	0.38 ± 0.50	0.42 ± 0.65	0.42 ± 0.73	0.376
TC, mmol/L	4.17 ± 1.09	4.18 ± 1.10	4.21 ± 1.08	4.10 ± 1.10	0.004
HDL-C, mmol/L	1.02 ± 0.28	1.01 ± 0.28	1.04 ± 0.28	0.99 ± 0.27	< 0.001
LDL-C, mmol/L	2.48 ± 0.92	2.50 ± 0.94	2.51 ± 0.92	2.41 ± 0.90	0.001
TG, mmol/L	1.81 ± 1.12	1.78 ± 1.09	1.75 ± 0.94	1.92 ± 1.37	< 0.001
Creatinine (μmol/L)	75.4 ± 16.0	75.6 ± 15.9	75.5 ± 15.2	75.0 ± 17.3	0.485
Lesion vessels	1.4 ± 0.7	1.4 ± 0.6	1.4 ± 0.7	1.5 ± 0.7	< 0.001
LM/three-vessel disease, %	2773 (45.2)	567 (40.7)	1224 (42.4)	982 (52.7)	< 0.001
SYNTAX score^a^	12.0 ± 8.2	11.7 ± 7.9	11.7 ± 8.1	12.6 ± 8.5	0.001
Complete revascularization, %	3310 (53.9)	798 (57.2)	1646 (57.1)	866 (46.5)	< 0.001
Number of stents	1.8 ± 1.2	1.8 ± 1.1	1.8 ± 1.1	1.9 ± 1.3	0.028
DES implantation, %	5778 (94.1)	1323 (94.9)	2730 (94.7)	1725 (92.6)	0.006
Medications at discharge, %					
Aspirin	6077 (99.0)	1372(98.4)	2859(99.1)	1846(99.1)	0.067
Clopidogrel	6020 (98.0)	1366(98.0)	2827(98.0)	1827(98.1)	0.959
β-blocker	5609 (91.4)	1250(89.7)	2622(90.9)	1737(93.3)	0.001
ACEI/ARB	3150 (51.3)	697(50.0)	1396(48.4)	1057(56.8)	< 0.001
CCB	2875 (46.8)	604(43.3)	1341(46.5)	930(49.9)	0.001
Statin	5923 (96.5)	1332(95.6)	2801(97.1)	1790(96.1)	0.022
Nitrate	5984 (97.5)	1358(97.4)	2820(97.8)	1806(97.0)	0.240

Values are presented as mean ± standard deviation or number (%)

*ACEI* angiotensin-converting enzyme inhibitors, *ACS* acute coronary syndrome, *ARB* angiotensin II receptor blockers, *BMI* body mass index, *CAD* coronary artery disease, *CABG* coronary artery bypass grafting, *CCB* calcium channel blocker, *DES* drug-eluting stent, *FIB* fibrinogen, *FBG* fasting blood glucose, *hs-CRP* high sensitivity C-reactive protein, *HbA1c* Hemoglobin A1c, *HDL-C* high-density lipoprotein cholesterol, *LDL-C* low-density lipoprotein cholesterol, *LM* left main disease, *LVEF* left ventricular ejection fraction, *MI* myocardial infarction, *PCI* percutaneous coronary intervention, *SAP* stable angina pectoris, *TC* total cholesterol, *TG* triglycerides

^a^Calculated using an online calculator (http://www.syntaxscore.com/) by a dedicated research group blinded to the clinical data

### Relationship between HbA1c/FBG and FIB

**Fig. 2:**
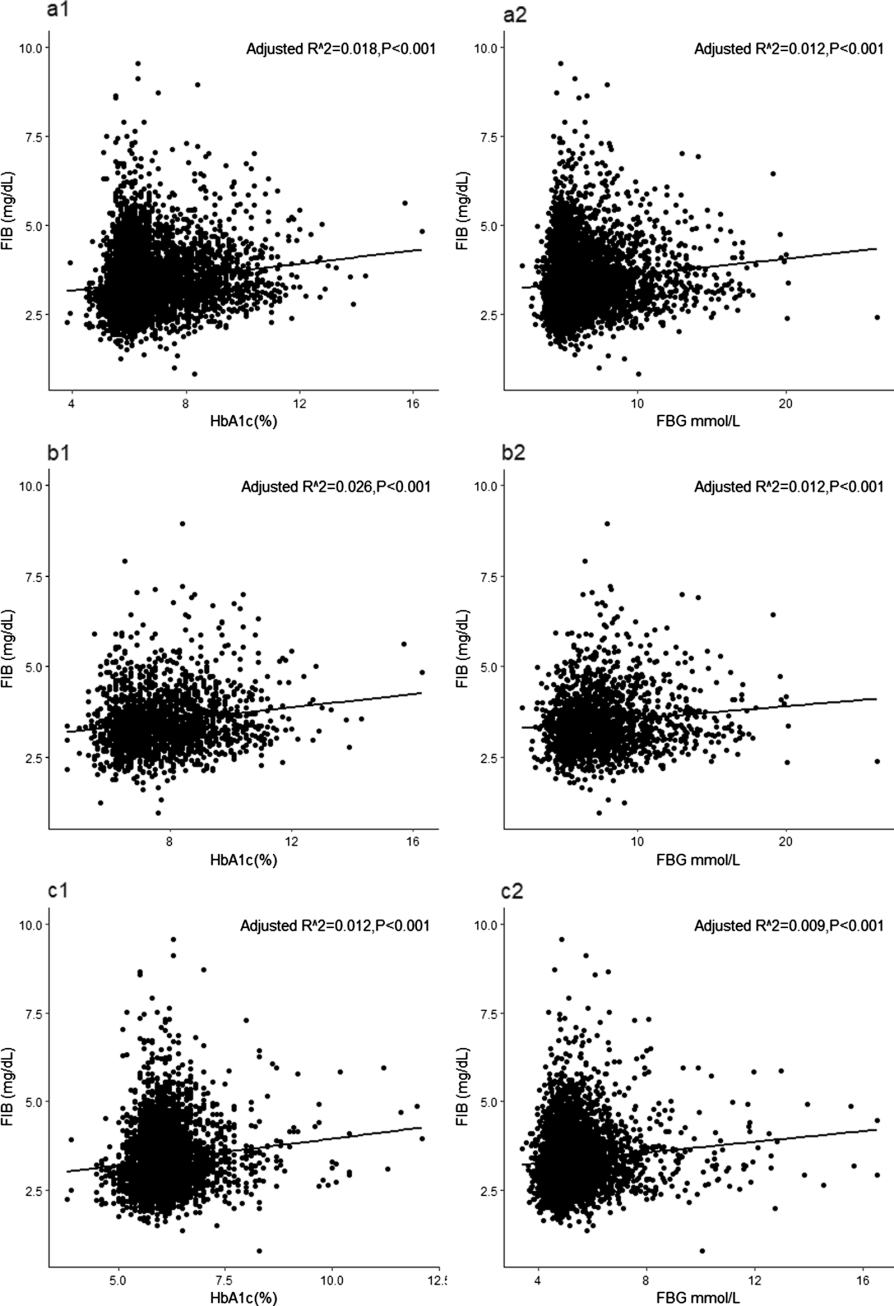
Correlation analysis of the relationship between glycemic metabolism and FIB. **a** Linear regression analysis of the relationship between glycemic metabolism (HbA1c and FBG) and FIB in whole patients. **b** Correlation analysis of the relationship between glycemic metabolism (HbA1c and FBG) and FIB in patients with DM. **c** Correlation analysis of the relationship between glycemic metabolism (HbA1c and FBG) and FIB in patients without DM. DM, diabetes mellitus, FBG, fasting blood glucose, FIB, fibrinogen, HbA1c, hemoglobin A1c

Linear regression analysis was used to assess the correlation between glycemic metabolism and FIB (Table 3). The results showed that both admission HbA1c (R^2^ = 0.018, Standard β = 0.133, P < 0.001) and FBG (R^2^ = 0.012, Standard β = 0.111,P < 0.001) were positively associated with FIB in the whole cohort. In DM patients, HbA1c (R^2^ = 0.026, Standard β = 0.163, P < 0.001) and FBG (R^2^ = 0.012, Standard β = 0.111,P < 0.001) were also positively associated with FIB. Furthermore, this positive relationship between HbA1c (R^2^ = 0.012, Standard β = 0.111, P < 0.001) and FBG (R^2^ = 0.009, Standard β = 0.096, P < 0.001) with FIB remained significant in the non-DM patients (Fig. 2). Of note, the correlation coefficients between HbA1c/FBG and FIB were relatively weak and may not be able to provide sufficient clinical value despite of statistically significant correlation.

**Table 3:** Correlation analysis between glycemic metabolism and FIB in patients with DM, without DM and whole

Variables	Adjusted R^2^	Coefficient	Standard β	SEM	*P* value
Whole					
HbA1c, %	0.018	0.133	0.133	0.009	< 0.001
FBG, mmol/L	0.012	0.111	0.111	0.005	< 0.001
DM					
HbA1c, %	0.026	0.162	0.163	0.013	< 0.001
FBG, mmol/L	0.012	0.111	0.111	0.007	< 0.001
Non-DM					
HbA1c, %	0.012	0.112	0.111	0.020	< 0.001
FBG, mmol/L	0.009	0.097	0.096	0.012	< 0.001

*DM* diabetes mellitus, *FBG* fasting blood glucose, *FIB* fibrinogen, *HbA1c* Hemoglobin A1c, *SEM* standard error of estimate

### Predictive value of FIB on all-cause mortality and cardiac mortality

The median follow-up time was 5.1 years (interquartile range 5.0–5.2 years), and the response rate was 91.2% (Fig. 1). During follow-up, 214 (3.5%) patients died, with 127 (59.3%) of whom from cardiac causes. Myocardial infarction (37.8%) was the most frequently reported cause of cardiac mortality, while malignancy (13.1%) was the most common cause of non-cardiac mortality (Additional file 1: Table S1).

**Fig. 3:**
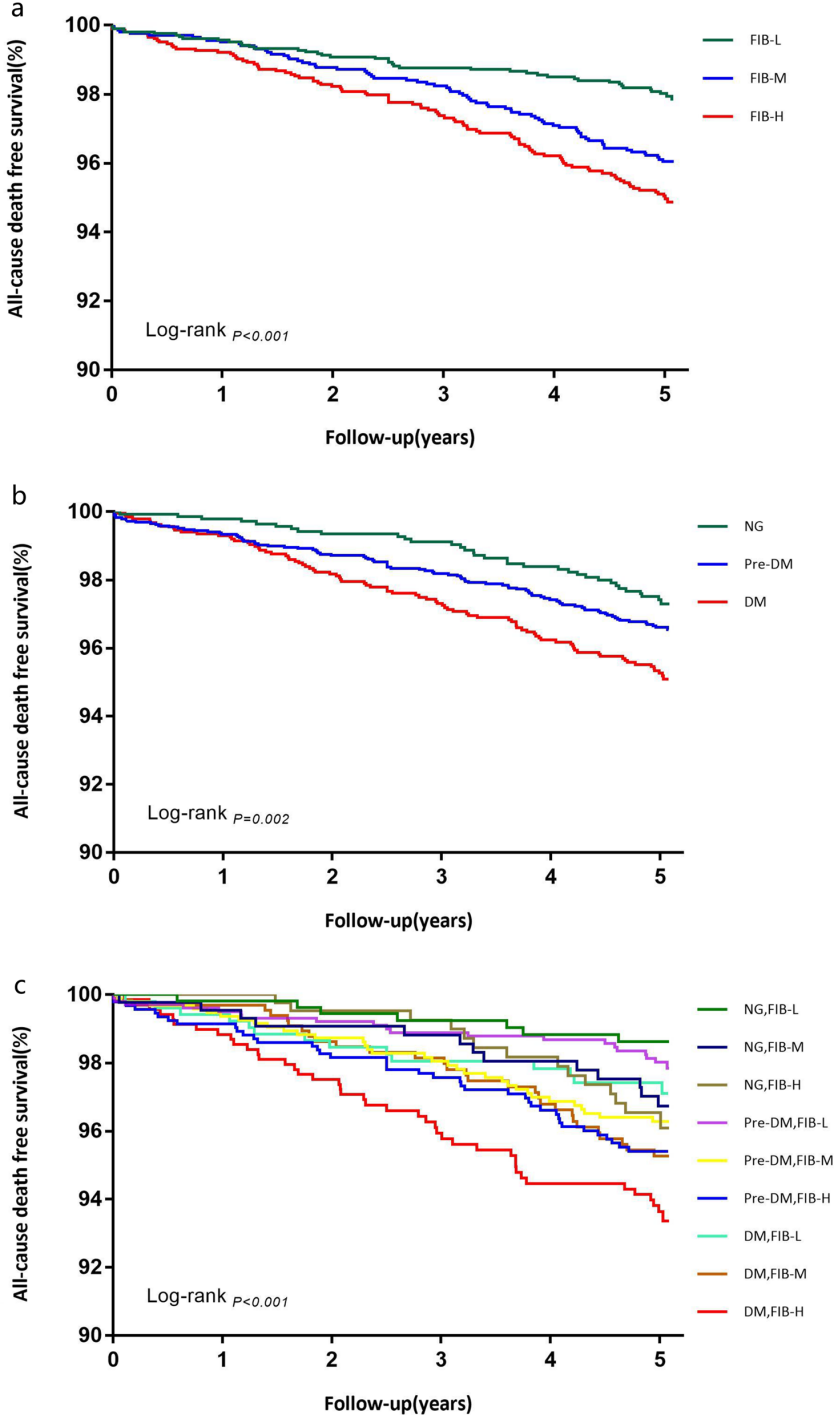
Kaplan–Meier analysis for all-cause death according to different FIB levels (**a**), glycemic metabolism status (**b**), and status of both FIB levels and glycemic metabolism (**c**)

The incidence of all-cause mortality in FIB-L, FIB-M and FIB-H group was 2.1%, 3.7% and 4.7%, respectively. Restricted cubic splines visualized a positive relation between FIB on a continuous scale with long-term risk of all-cause mortality and cardiac mortality (all P for non-linearity > 0.05) (Additional file 1: Figure S1). The Kaplan–Meier survival curve revealed that patients with higher FIB levels had significantly increased risk of all-cause mortality and cardiac mortality (all log-rank P < 0.001) (Fig. 3a, Additional file 1: Figure S2a). The univariate Cox analysis showed a strong relation between continuous FIB with all-cause mortality (HR: 1.36; 95% CI 1.20–1.55 per 1 unit increase in FIB; P < 0.001) and cardiac mortality (HR: 1.46; 95% CI 1.25–1.71 per 1 unit increase in FIB; P < 0.001). On multivariate analysis, the relationship between continuous as well as tertiles of FIB with all-cause mortality (adjusted HR: 1.23; 95% CI 1.07–1.42 per 1 unit increase in FIB; P = 0.004) and cardiac mortality (adjusted HR: 1.31; 95% CI 1.10–1.55 per 1 unit increase in FIB; P = 0.003) remained statistically significant after adjustment for potential confounders (Table 4).

**Table 4:** Predictive value of the FIB level for all-cause death and cardiac death in univariate and multivariate analysis

Variables	Events/subjects	Univariate analysis		Multivariate analysis	
		Hazard ratio (95% CI)	P value	Hazard ratio (95% CI)	P value
All-cause death	214/6140				
FIB (per 1-unit increase)		1.36 (1.20–1.55)	< 0.001	1.23 (1.07–1.42)	0.004
FIB-L	43/2069	Reference	–	Reference	–
FIB-M	75/2033	1.81 (1.24–2.63)	0.002	1.72 (1.18–2.51)	0.005
FIB-H	96/2038	2.35 (1.64–3.36)	< 0.001	1.86 (1.28–2.69)	0.001
Cardiac death	127/6140				
FIB (per 1-unit increase)		1.46 (1.25–1.71)	< 0.001	1.31 (1.10–1.55)	0.003
FIB-L	28/2069	Reference	–	Reference	–
FIB-M	36/2033	1.33 (0.81–2.18)	0.259	1.26 (0.76–2.07)	0.369
FIB-H	63/2038	2.36 (1.51–3.68)	< 0.001	1.82 (1.15–2.89)	0.011

Model adjusted for age, sex, BMI, hypertension, diabetes, family history of CAD, prior PCI/CABG, LVEF, LDL-C, creatine, DES implantation, clopidogrel, ACEI/ARB

*CI* confidence interval, *FIB* fibrinogen

### Glycemic metabolism, FIB levels and occurrence of all-cause mortality

**Fig. 4:**
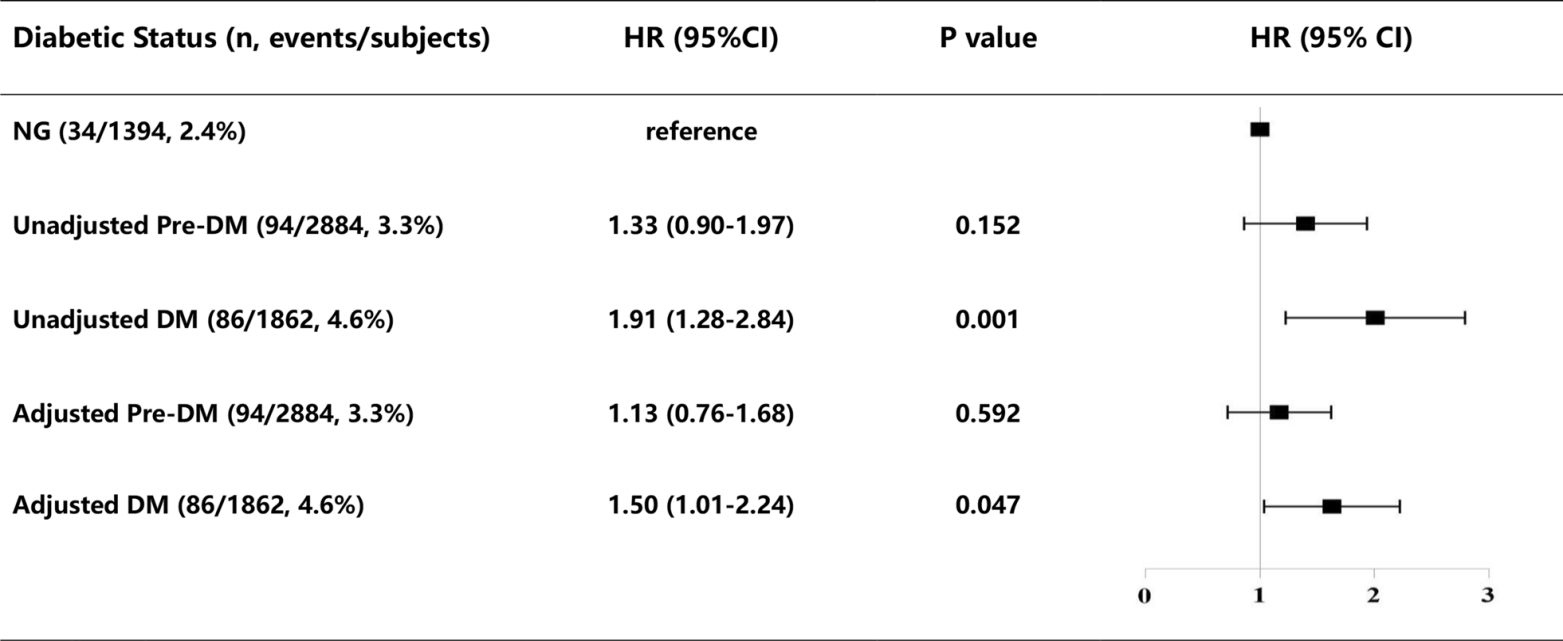
Relations of different glycemic metabolism status and all-cause death in univariate and multivariate survival analysis. Model adjusted for age, sex, BMI, hypertension, family history of CAD, prior PCI/CABG, LVEF, LDL-C, creatine, DES implantation, clopidogrel, ACEI/ARB. CI, confidence interval; NG, normoglycemia, Pre-DM, prediabetes; DM, diabetes mellitus

The prevalence of all-cause mortality in NG, Pre-DM and DM group was 2.4%, 3.3% and 4.7%, respectively. The Kaplan–Meier curve demonstrated that patients with DM had significantly increased risk of all-cause mortality and cardiac mortality among the three groups (all log-rank P < 0.05) (Fig. 3b, Additional file 1: Figure S2b). Univariate Cox analysis revealed that DM group had 1.91-fold higher risk of all-cause mortality (HR: 1.91; 95% CI 1.28–2.84; P = 0.001) and 2.18-fold higher risk of cardiac mortality (HR: 2.18; 95% CI 1.28–3.37; P = 0.004) when compared with NG group. And this significant association remained unchanged after adjustment for other covariates. However, Pre-DM group did not increase the risk of all-cause mortality and cardiac mortality compared with NG group (Fig. 4, Additional file 1: Figure S3).

When patients were evaluated according to both glycemic metabolism and FIB levels, the Kaplan–Meier curve showed that those with DM and FIB-H levels had significantly highest risk of all-cause mortality compared with the reference group (NG plus FIB-L group, log rank P < 0.001). Furthermore, NG plus FIB-H, Pre-DM plus FIB-M, Pre-DM plus FIB-H and DM plus FIB-M groups also had significantly increased risk of all-cause mortality than the reference group (NG plus FIB-L group, all log rank P < 0.05) (Fig. 3c). The further univariate Cox analysis revealed similar results. Multivariate Cox analysis according to both glycemic metabolism and FIB levels indicated that patients in Pre-DM plus FIB-H, DM plus FIB-M and DM plus FIB-H groups had 2.27-fold (adjusted HR: 2.27; 95% CI 1.01–5.12), 2.57-fold (adjusted HR: 2.57; 95% CI 1.12–5.89) and 3.04-fold (adjusted HR: 3.04; 95% CI 1.35–6.82) higher risk of all-cause mortality, respectively (all P < 0.05) (Table 5).

**Table 5:** Relation of the FIB level and all-cause death in patients with different glycemic metabolism status

Variables	Events/subjects	HR (95% CI)	
	214/6140	Crude model	Adjusted model
NG			
FIB-L	7/537	Reference	Reference
FIB-M	13/433	2.35 (0.94–5.87)	2.08 (0.83–5.22)
FIB-H	14/424	2.62 (1.06–6.49) *	1.96 (0.79–4.89)
Pre-DM			
FIB-L	21/1011	1.59 (0.68–3.74)	1.24 (0.53–2.93)
FIB-M	33/944	2.73 (1.21–6.12) *	2.17 (0.96–4.93)
FIB-H	40/929	3.40 (1.52–7.58) **	2.27 (1.01–5.12) *
DM			
FIB-L	15/521	2.23 (0.91–5.46)	1.67 (0.68–4.10)
FIB-M	29/656	3.45 (1.51–7.89) **	2.57 (1.12–5.89) *
FIB-H	42/685	4.91 (2.20–10.92) **	3.04 (1.35–6.82) **

Model adjusted for age, sex, BMI, hypertension, diabetes, family history of CAD, prior PCI/CABG, LVEF, LDL-C, creatine, DES implantation, clopidogrel, ACEI/ARB

*CI* confidence interval, *FIB* fibrinogen, *NG* normoglycemia, *Pre-DM* prediabetes, *DM* diabetes mellitus

* P value < 0.05, ** P value < 0.01

## Discussion

Using a large, real-world, prospective cohort sample, we found that FIB positively correlated with glycemic metabolism in CAD patients undergoing PCI. Moreover, higher FIB levels, analyzed as continuous or categorical variables, were strongly associated with increased risk of long-term all-cause and cardiac mortality. Furthermore, poorer long-term outcomes were also found in diabetic patients, but not in prediabetic patients. Interestingly, when patients were categorized into 9 groups according to both FIB levels and glycemic metabolism status, patients with pre-DM plus high FIB levels, DM plus medium FIB levels and DM plus high FIB levels had increased risk of all-cause mortality than those with NG and low FIB levels. For the first time, our study demonstrated that FIB might affect the long-term prognosis in CAD patients with pre-DM undergoing PCI, and indicated a joint prognostic value of FIB levels and impaired glycemic metabolism on mortality in CAD patients undergoing PCI.

FIB is a crucial glycoprotein consisting of three different polypeptides, which is mainly synthesized in the liver^15^. Upon action of thrombin, FIB is transformed into fibrin monomer which then crosslinks platelets, increases blood viscosity and ultimately leads to clot formation^3^. Besides, FIB levels are elevated in response to various chronic inflammatory conditions, including DM, obesity and atherosclerosis^7^,^ 12^,^ 16^. It is also directly involved in the pathogenesis of atherosclerosis through multiple mechanisms, such as inducing endothelial dysfunction, stimulating smooth muscle cell proliferation and migration, facilitating monocyte or macrophage adhesion and infiltration of atherosclerotic lesions, which will jointly potentiate plaque evolution^17^.

To date, studies have been conducted on the prognostic value of FIB in different clinical settings. Aside from the positive association with all-cause and CVD mortality in general individuals^6^,^ 18^,^ 19^, FIB was reported to be an independent risk factor of the occurrence and severity of CAD^20^. Further, both small sample and large epidemiological studies showed that FIB was associated with worse clinical outcomes in patients with stable CAD^10^,^ 21^,^ 22^. A recent prospective study from China indicated elevated FIB was also strongly associated with MACE risk in ACS patients, especially when complicated with DM^9^. Similarly, the present study found FIB had an independent association with long-term all-cause and cardiac mortality in CAD patients undergoing PCI. Instead, the ADVANCE study including 3,865 diabetic patients showed FIB was not an independent predictor of 5-year mortality. It is worth noting that only limited number of patients in the ADVANCE study underwent coronary revascularization, while in our study all patients were treated with PCI [23]. The PRIME study including 926 men aged 50 to 59 without CAD found that FIB was not a risk marker of MI-coronary death. The differences in endpoints and sample size might contribute to the difference in results between this study and ours^24^. The EPIC-Norfolk study including 16,850 participants who were free of cancer, MI and stroke at baseline found that FIB was not a predictor of all-cause and cardiovascular mortality. However, the data used in the EPIC-Norfolk cohort are rather old and the serum used for measuring FIB and other biomarkers was stored frozen for more than ten years, which might limit the reliability of the results 25. Notably, none of these studies focused only on CAD population. This may be another possible explanation for the controversy between these studies and our findings. In spite of the conflicting findings mentioned above, data from the latest clinical trials confirmed the benefit of anti-inflammatory effect both in patients with chronic coronary disease and acute MI^26^,^ 27^. Considering the role of FIB as an inflammatory biomarker, additional studies are warranted to further evaluate whether FIB could be helpful to identify high-risk individuals in CAD patients.

Currently, glycemic metabolism abnormality including DM and pre-DM is prevalent in clinical practice, especially in patients with established CAD^11^. It has been previously demonstrated that DM independently increased the risk of adverse CVD events in CAD patients^28^. Notably, CAD patients with pre-DM seemed to share similar clinical outcomes with those with normoglycemia^10^,^ 29^. However, when combined with other disorders, such as dyslipidemia or hypertension, prediabetic patients with CAD were demonstrated to have significantly less favorable prognosis^30^–^32^. Interestingly, a large-sample observational study recently reported that elevated FIB increased the MACE risk in patients with stable CAD only in the presence of DM and pre-DM, indicating FIB to be valuable for prognostic assessment in prediabetic patients with stable CAD^10^. However, the combined value of FIB and impaired glycemic metabolism on prediction of mortality in CAD patients undergoing PCI is still unclear. In this study, we observed that among CAD patients undergoing PCI, diabetic individuals with medium or high FIB levels had 2.57-fold and 3.04-fold higher risk of mortality respectively during a median follow-up of 5.1 years. Furthermore, prediabetic patients also had higher mortality risk in the subgroup of high FIB levels, indicating that FIB may be useful for further risk stratification in CAD patients with mild impaired glycemic metabolism after PCI.

Patients with DM were confirmed to have higher levels of plasma FIB^12^. Chronic mild inflammation is a recognized pathological mechanism of DM^33^. Elevated FIB existing in diabetic patients aggravates the inflammatory process and the burden of atherosclerosis^4^,^ 34^. FIB also involves in insulin resistance and impair the normal glycemic regulation^13^. Moreover, elevated FIB could weaken platelet inhibition with clopidogrel in the presence of DM^35^. And this effect is mediated through its direct interaction with the GP IIb/IIIa receptor, which is independent from inflammation. Indeed, our study found that the average levels of FIB elevated from NG, pre-DM to DM. Moreover, FIB was also positively associated with glycemic metabolism (HbA1c and FBG) both in CAD patients with or without DM, which was basically consistent with the prior studies^9^,^ 10^. Collectively, although without established causality, the present study revealed a significant association between FIB and glycemic metabolism, as well as the long-term mortality in CAD patients undergoing PCI. Given the relatively simple and cost-effective test of FIB, these findings encourage its potential value as a biomarker in this specific population to identify high-risk patients, especially in those with DM and pre-DM. Meanwhile, the importance of routine screening for impaired glycemic metabolism also cannot be neglected.

Another issue to be discussed is the potential significance of lowering FIB levels in this specific population. Previous evidence showed the contribution of some lifestyle factors such as smoking, sedentary behavior and unhealthy diet to the elevation of plasma FIB levels^36^–^38^. On the contrary, exercise training significantly reduced FIB levels and improved cardiorespiratory fitness^39^. Therefore, lifestyle modification for patients with high FIB levels to achieve clinically favorable outcomes may be a reasonable exploration. In addition to plasmin, several medications such as fibrates are known to reduce FIB levels as an additional effect^40^. However, to date, no medication can specifically reduce FIB levels in the long run. Future studies are warranted to investigate whether patients could benefit from pharmacologic intervention on the premise that specific drug targeting FIB on a long-term basis is discovered.

This study has some limitations. First, FIB data at baseline was not available in about 40% patients, which might impair the strength of our study. Second, glycemic evaluation to identify new-onset diabetes and prediabetes was not routinely performed during follow-up. Third, information on other coagulation parameters such as prothrombin time (PT), activated partial thromboplastin time (APTT) and thrombin time (TT) were not collected previously. Fourth, the study period was relatively short and nearly 10% of patients were lost to follow-up. A longer follow-up such as ten years would be beneficial. Fifth, due to the observational design, potential confounders cannot be fully controlled.

## Conclusions

FIB was independently associated with long-term all-cause and cardiac mortality among CAD patients undergoing PCI, especially in those with DM and Pre-DM. FIB test may help to identify high-risk individuals in this specific population.

## Supplementary Information

**Additional file 1: Table S1.** Summary of cause of mortality. **Figure S1.** Restricted cubic splines of FIB levels in relation to relative hazard ratio for all-cause death and cardiac death. **Figure S2.** Kaplan-Meier analysis for cardiac death according to different FIB levels and glycemic metabolism status. **Figure S3.** Relations of different glycemic metabolism status and cardiac death in univariate and multivariate survival analysis.

## Data Availability

Due to ethical restrictions related to the consent given by subjects at the time of study commencement, our datasets are available from the corresponding author upon reasonable request after permission of the Institutional Review Board of Fuwai Hospital.

## Abbreviations

FIB: Fibrinogen
CAD: Coronary artery disease
PCI: Percutaneous coronary intervention
DM: Diabetes mellitus
Pre-DM: Prediabetes
NG: Normoglycemia
HbA1c: Hemoglobin A1c
FBG: Fasting blood glucose
CVD: Cardiovascular disease
ACS: Acute coronary syndrome
LDL-C: Low-density lipoprotein cholesterol
CI: Confidence interval
BMI: Body mass index
CABG: Coronary artery bypass grafting
LVEF: Left ventricular ejection fraction
DES: Drug-eluting stent
ACEI: Angiotensin-converting enzyme inhibitors
ARB: Angiotensin II receptor blockers
hs-CRP: high sensitivity C-reactive protein
TC: Total cholesterol
TG: Triglycerides
HDL-C: High-density lipoprotein cholesterol
MACE: Major adverse cardiovascular events
MI: Myocardial infarction
