# Supplementary material for: Prognostic value of fibrinogen in patients with coronary artery disease and prediabetes or diabetes following percutaneous coronary intervention: 5-year findings from a large cohort study
Source: Cardiovasc Diabetol. 2021 Jul 16;20:143. doi: 10.1186/s12933-021-01335-1 (PMC8283976; doi:10.1186/s12933-021-01335-1)
Supplement: Supplementary file 1 — Additional file 1: Table S1. Summary of cause of mortality. Figure S1. Restricted cubic splines of FIB levels in relation to relative hazard ratio for all-cause death and cardiac death. Figure S2. Kaplan-Meier analysis for cardiac death according to different FIB levels and glycemic metabolism status. Figure S3. Relations of different glycemic metabolism status and cardiac death in univariate and multivariate survival analysis. [file 12933_2021_1335_MOESM1_ESM.docx]

**Additional file 1**

**Table S1.** Summary of cause of mortality.

|  | Number |
| --- | --- |
| Overall death | 214 |
| Cardiac death | 127 (59.3%) |
| Myocardial infarction | 81 (37.8%) |
| Heart failure | 32 (14.9%) |
| Sudden cardiac death | 10 (4.6%) |
| Malignancy arrhythmia | 1 (0.5%) |
| Cardiac rupture | 1 (0.5%) |
| Cardiac procedure/operation-related death | 1 (0.5%) |
| Aortic aneurysm rupture | 1 (0.5%) |
| Non-cardiac death | 87 (40.7%) |
| Malignancy | 28 (13.1%) |
| Stroke | 24 (11.2%) |
| Infectious disease | 6 (2.8%) |
| Trauma | 4 (1.9%) |
| Multiple organ failure | 4 (1.9%) |
| Pulmonary failure | 3 (1.4%) |
| Diabetic complication | 2 (0.9%) |
| Renal failure | 2 (0.9%) |
| Bleeding | 1 (0.5%) |
| Peripheral artery disease | 1 (0.5%) |
| Undetermined cause | 12 (5.6%) |

**
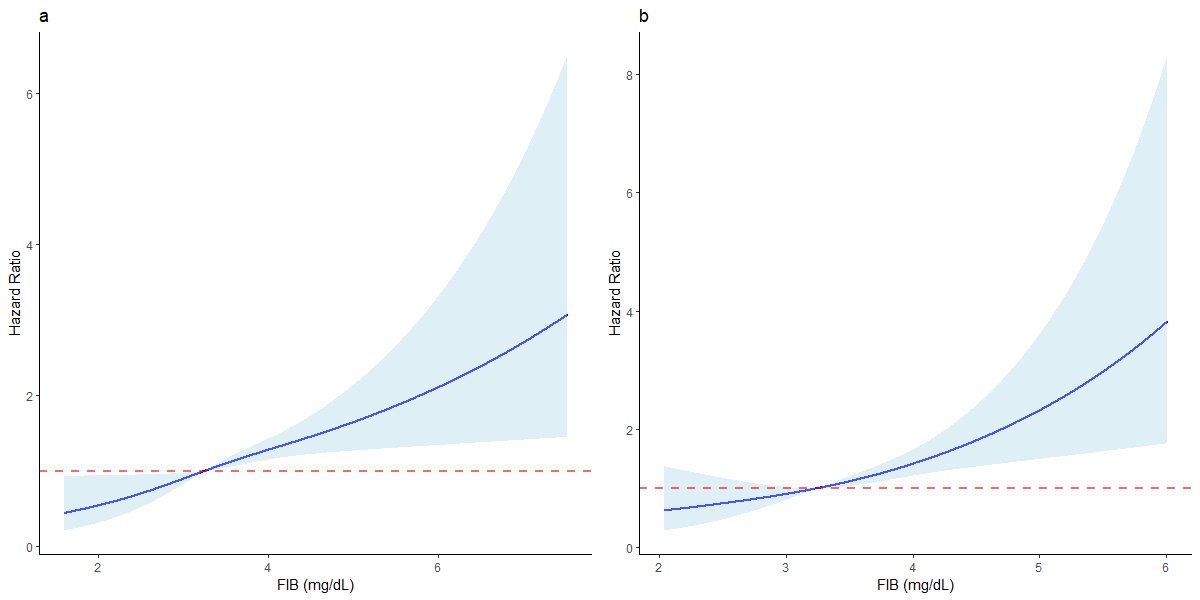
**

**Figure S1.** Restricted cubic splines of FIB levels in relation to relative hazard ratio (blue line with 95% confidence interval shaded in light blue) for the all-cause death (**a**) and cardiac death (**b**). Red lines, reference. FIB, fibrinogen.

**
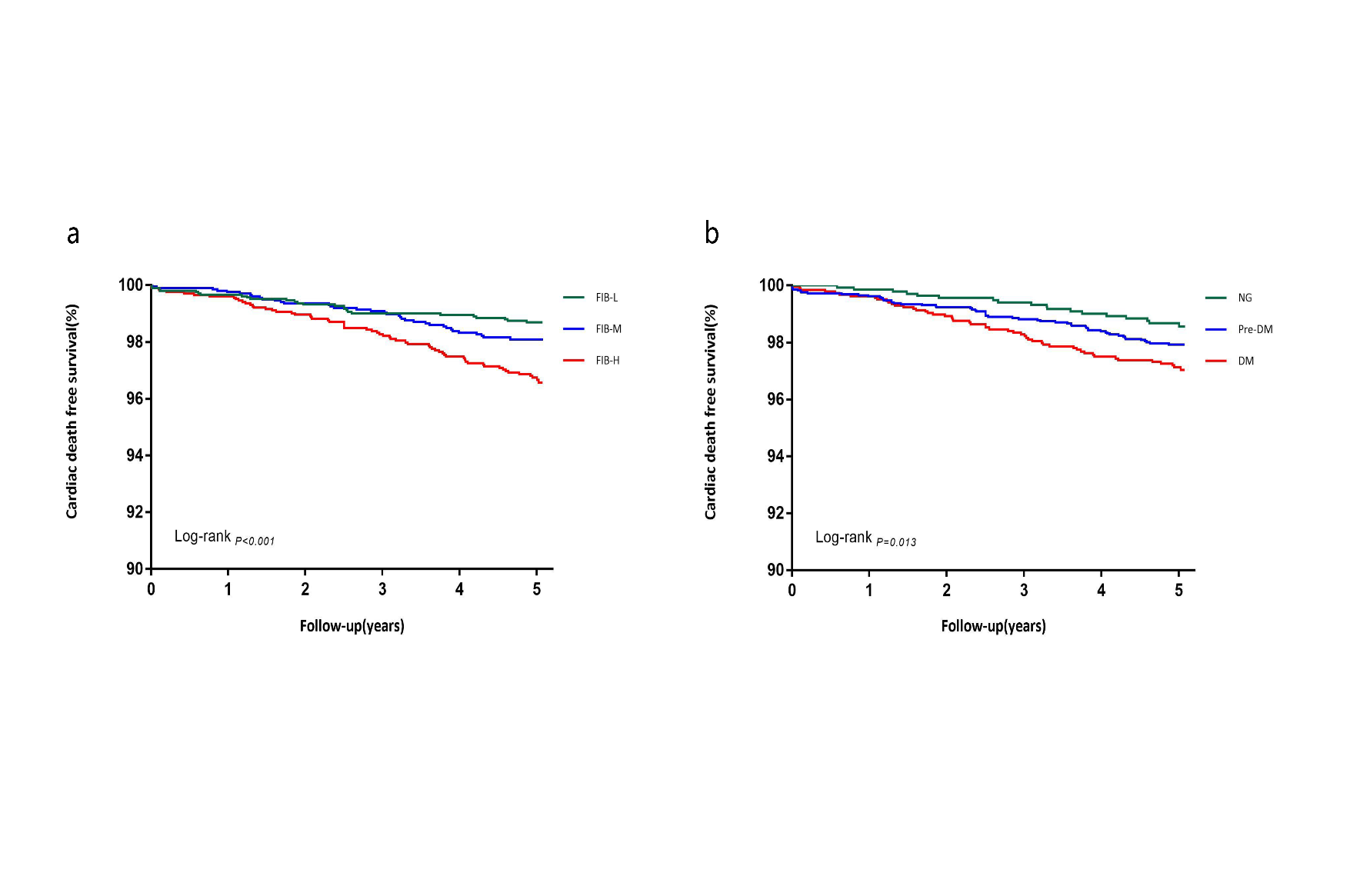
**

**Figure S2.** Kaplan-Meier analysis for cardiac death according to different FIB levels (**a**), glycemic metabolism status (**b**).

**
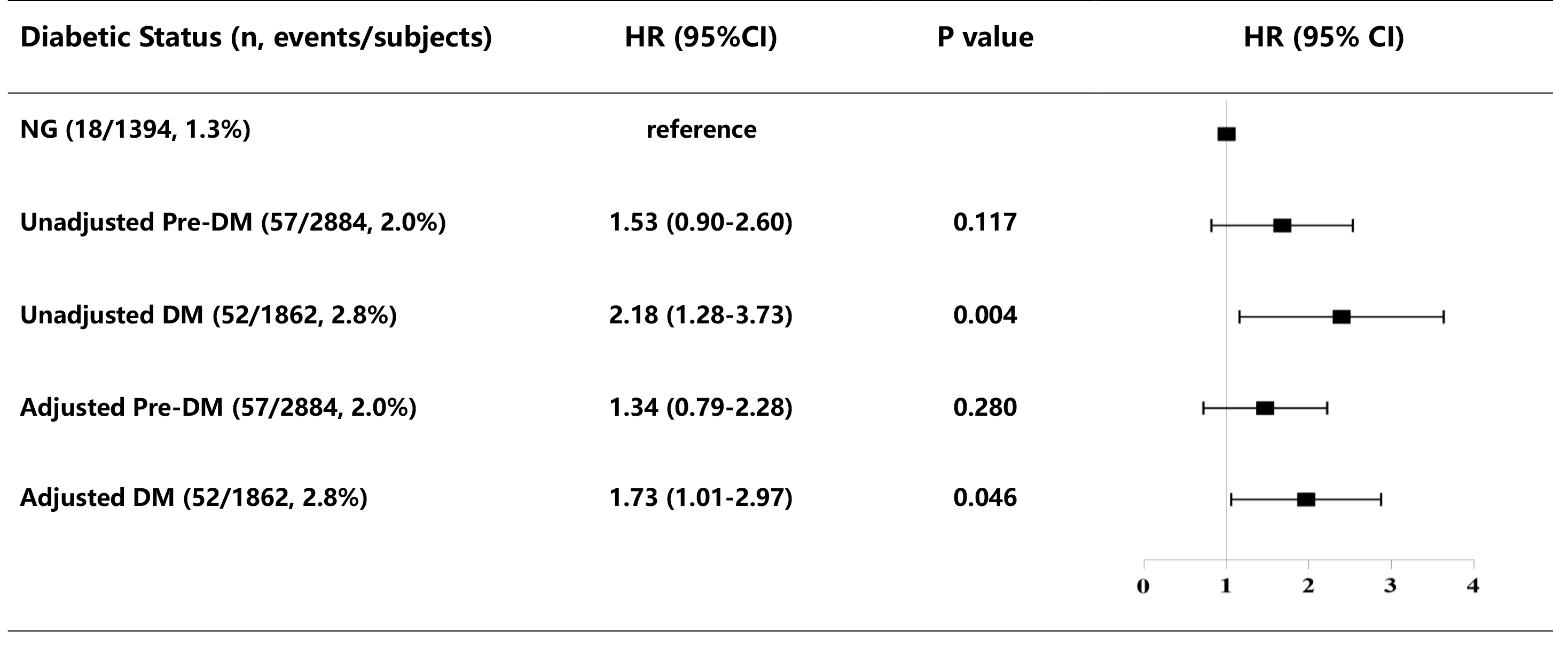
**

**Figure S3.** Relations of different glycemic metabolism status and cardiac death in univariate and multivariate survival analysis. Model adjusted for age, sex, BMI, hypertension, family history of CAD, prior PCI/CABG, LVEF, LDL-C, creatine, DES implantation, clopidogrel, ACEI/ARB. CI, confidence interval; NG, normoglycemia, Pre-DM, prediabetes; DM, diabetes mellitus.
